# Replication Pilot Trial of Therapeutic Horseback Riding and Cortisol Collection With Children on the Autism Spectrum

**DOI:** 10.3389/fvets.2018.00312

**Published:** 2019-01-14

**Authors:** Zhaoxing Pan, Douglas A. Granger, Noémie A. Guérin, Amy Shoffner, Robin L. Gabriels

**Affiliations:** ^1^Department of Pediatrics, University of Colorado Anschutz Medical Campus, Aurora, CO, United States; ^2^Children's Hospital Colorado, Aurora, CO, United States; ^3^Institute for Interdisciplinary Salivary Bioscience Research, University of California, Irvine, Irvine, CA, United States; ^4^Bloomberg School of Public Health and School of Nursing, Johns Hopkins University School of Medicine, Baltimore, MD, United States; ^5^Center for the Human-Animal Bond of Purdue, College of Veterinary Medicine, Purdue University, West Lafayette, IN, United States

**Keywords:** autism spectrum disorder, equine-assisted activities and therapies, human-animal interaction, therapeutic horseback riding, salivary cortisol

## Abstract

We aimed to determine whether results of our prior randomized control trial [RCT; NCT02301195, (1)] of Therapeutic Horseback Riding (THR) for children and adolescents with autism spectrum disorder (ASD) could be *replicated* at a different riding center and if treatment effects also included differences in the expression of associations between problem behavior and the activity of the hypothalamic-pituitary-adrenal (HPA) axis. Participants with ASD (*N* = 16) ages 6-16 years were randomized by nonverbal intelligence quotient to either a 10-week THR group (*n* = 8) or no horse interaction barn activity (BA) control group (*n* = 8). Outcome measures were a standard speech-language sample and caregiver-report of aberrant and social behaviors. Participants' saliva was sampled weekly at a consistent afternoon time immediately pre- and 20 min' post-condition (later assayed for cortisol). Intent-to-treat analysis revealed that compared to controls, THR participants had significant improvements in hyperactivity, and social awareness, and significant improvements at the 0.1 significance level in irritability and social communication behaviors. There were no significant improvements in number of words or new words spoken during the standard language sample. Linear mixed effects model analysis indicated that greater weekly pre-lesson irritability levels were associated with smaller post-lesson reduction in salivary cortisol levels, and greater weekly pre-lesson hyperactivity levels were associated with smaller cortisol reduction in the THR group, but not in the BA control group. The findings represent a partial replication of prior results (1), extend prior observations to include THR effects on biobehavioral relationships and suggest that cortisol could be a target mediator for THR effects on irritability and hyperactivity behaviors in youth with ASD.

**Clinical Trial Registration:** Trial of Therapeutic Horseback Riding in Children and Adolescents with Autism Spectrum Disorder; http://clinicaltrials.gov, identifier: NCT02301195

## Introduction

In addition to core impairments in social and communication skills, restricted interests, and repetitive behaviors (2), individuals with autism spectrum disorder (ASD) have high rates of co-existing psychiatric symptoms that include anxiety, depression, irritability, and attention-deficit and hyperactivity disorder (3–11). Such co-existing conditions can impair functioning, which puts this population at risk to engage in dangerous aberrant behaviors (12) (e.g., aggression and self-injury) and to seek costly crisis psychiatric care services (e.g., emergency department and inpatient hospitalization) (13, 14). To proactively address the core impairments and aberrant behaviors unique to individuals with ASD, one increasingly popular intervention is animal-assisted intervention (AAI) (15, 16).

Systematic reviews of the literature reflect a recent increase in the quantity and quality of research on AAI with the pediatric population of individuals with ASD (15, 16). Most studies of AAI programs for ASD are comprised of 8–12 weekly sessions, and the most commonly reported outcome is improved social interactions. Horses are the most common species included in AAI research through the practice of therapeutic horseback riding (THR) (16); In 2015, Gabriels et al. conducted the first large-scale randomized clinical trial of THR for children with ASD, with 127 participants ages 6–16 (1). Compared to participants in a barn activity (BA) control group, participants in a 10-week THR intervention made significant improvements in symptoms of irritability and hyperactivity as measured by the Aberrant Behavior Checklist-Community (ABC-C) (17), improvements in core symptoms of autism (e.g., social cognition and social communication) measured by the Social Responsiveness Scale (SRS) (18), and word fluency (e.g., total number of words and new words spoken) measured by a standardized language sample (1). A more recent study of THR replicated use of the ABC-C (17) to measure outcomes in a sample of 26 children with ASD (19). This study found that children participating in five to seven 45-minute weekly riding lessons compared to a control group receiving treatment as usual, improved on the ABC-C (17) Hyperactivity scale, but not on the Irritability scale (19). It is promising that Harris and Williams (19) attempted to replicate the irritability and hyperactivity outcomes previously observed by Gabriels et al. (1); however, the advancement of the AAI field requires more methodological standardization and replication of methods to confirm the efficacy of THR on outcomes in children with ASD (15, 16, 20, 21). Additionally, improved methodological rigor can lead to an increased understanding of the mechanisms, such as physiological arousal levels, that might help explain observed benefits of, for example, THR on children with ASD.

The field of AAI has historically claimed that interacting with animals can reduce an individual's arousal level to dampen stressed/anxious states. There are a number of AAI studies that have observed favorable autonomic response patterns using physiological measures (e.g., cortisol, cardiovascular, electrodermal) in individuals when they are engaged with animals, providing support for the assertion that AAI can produce a regulated state of arousal (22).

In the ASD population, poorly regulated emotional/arousal states tend to manifest as symptoms of stress/anxiety, depression, irritability, and hyperactivity, which are particularly prevalent (11, 23). Specifically, irritability behaviors in the ASD population have been characterized as heightened emotional (e.g., anger) and behavioral (e.g., aggression. severe tantrums, self-injury) reactivity (24), behaviors that often require high levels of intensive interventions. Given this information, it is reasonable to hypothesize that elements inherent in THR may activate a physiological state of regulation that leads to beneficial outcomes such as reductions in irritability behaviors.

Our understanding of the effects of AAI on physiological arousal levels such as the reactivity and regulation of environmentally sensitive biological systems, such as the hypothalamic-pituitary-adrenal (HPA) axis, and the association of these AAI-related changes in physiology with behavior in the context of ASD is in its infancy.

The HPA axis is one of the two main components of the psychobiology of the stress response, and its primary product, cortisol can accurately (using minimally invasive collection methods) be measured in saliva. An extensive literature reveals changes in cortisol in response to novelty, defeat, and social evaluative threat and these changes are most pronounced when individuals do not have prior experience or sufficient coping skills or resources to adapt to those events by changing their actions or thoughts [see for review (25)]. A 2014 review of cortisol investigations in the ASD population, reported that individuals with high rates of irritability behaviors show a more sluggish response of the HPA axis to stressors (26). A similar finding was reported in a study of high functioning (HF) boys with ASD who endorsed having high levels of irritability, yet their cortisol levels were lower/less responsive to a psychosocial stress test compared to HF boys with ASD who endorsed having lower levels of irritability (27). These recent study findings raise questions about the role of irritability in influencing the physiological response patterns (e.g., HPA axis) in the ASD population.

The handful of studies of HPA axis reactivity and regulation in ASD suggest that compared to typically-developing children, children with ASD experience higher HPA axis reactivity to daily stressors (28, 29). Understanding whether the effects of AAI reveal at both the behavioral surface, and the level of fast acting environmentally sensitive biological systems, like the HPA axis, may be key to advancing our understanding of individual differences in, or the degree of short- versus longer-term, benefits of AAI in the context of ASD. An RCT on typically-developing adolescents found that compared to a control group, adolescents participating in an 11-week equine-facilitated learning (EFL) program had lower basal salivary cortisol levels (30). One study examining the effect of service dogs on salivary cortisol levels of 42 children with ASD found that having a service dog led to significantly lower cortisol awakening responses (CAR), but did not influence average diurnal cortisol levels (31). In eight male children with ASD, hippotherapy led to reduced cortisol after riding compared to before riding, apart from the first riding session, which may represent the stressful effect of getting used to a new environment and riding for the first time (32). Overall, it seems that AAI may have a direct, at least short term, effect on reactivity and regulation of the HPA axis.

In the present study, the first aim was to implement a previously reported THR intervention model from a large scale RCT in a different THR riding center to examine its feasibility and effectiveness (1). The second aim was to extend the findings of Gabriels et al. (1) by replicating effects of THR on ASD-related aberrant behavior, but also by examining treatment effects on levels of cortisol before and 20 min after THR, and on the expression of the association between cortisol and ASD-related aberrant behavior.

## Materials and Methods

### Participants

**Figure 1:**
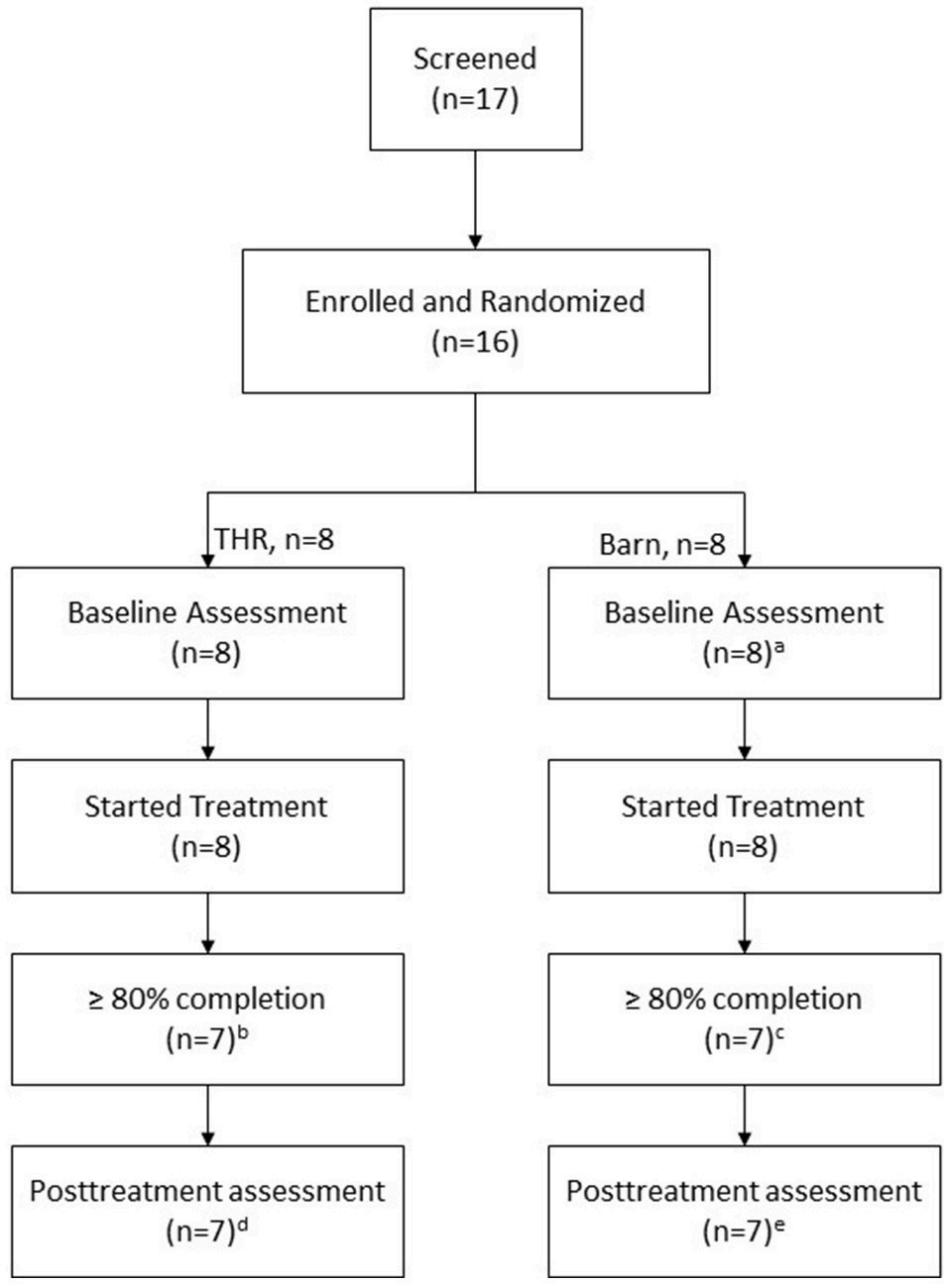
Screening, enrollment, randomization, and follow-up of study participants. ^a^ABC-C was not returned for one participant. ^b^One participant completed 5 sessions only. ^c^One participant completed 2 sessions only. ^d^Of the 7 participants, one has no posttreatment SALT evaluation but ABC-C and SRS. ^e^Of the 7 participants, one has no posttreatment ABC-C data but SALT and SRS.

For this IRB-approved study, participants were recruited via inpatient hospital and out-patient therapy services, schools, and ASD-parent groups. Participant inclusion criteria replicated those reported by Gabriels et al. (1): Ages 6–16 years; a diagnosis of ASD confirmed [i.e., meeting the cut-off of ≥15 on the Social Communication Questionnaire (SCQ) (33) and meeting the empirically-derived cutoffs for ASD or Autism on the Autism Diagnostic Observation Schedule-2nd Edition (ADOS-2) (34)]; a combined total score of >11 on the Irritability and Stereotypy subscales of the Aberrant Behavior Checklist-Community (ABC-C) (17); and a nonverbal IQ (NVIQ) score of ≥40 standard score measured by the Leiter-3 (35). Exclusion criteria also included a screening for contraindications based on guidelines from the Professional Association of Therapeutic Horsemanship International (PATH Intl.) *Standards for Certification and Accreditation* (36). Contraindications included medical or behavioral concerns that might make it dangerous to participate in the horseback riding activity such as uncontrolled seizures, or a history of animal abuse. Participants were also excluded if they had participated in a THR intervention within 6 months prior to entering the study, weighed 200 pounds or more, exceeding the riding center's policies to ride a horse, or if they were taking steroid medications, as steroids might confound cortisol results. See Figure 1 for screening and enrollment information.

### Study Design

#### Screening Visit I

Interested caregivers and participants were engaged in an IRB-approved informed consent/assent and screening process at the first authors' institution setting before traveling to the riding center for a second level screening. During this first screening, caregivers completed demographic, diagnostic and behavior rating forms regarding their child that included the SCQ (33), ABC-C (17) and the Spence Children's Anxiety Scale-Parent Version (SCAS-P) (37). Participants completed the Leiter-3 (35) and ADOS-2 (34). Additionally, participants and their caregivers were instructed (via demonstration and hands-on practice) how to collect saliva samples, provided with visual food cues to help stimulate saliva production and informed that the child participant needed to avoid eating, drinking or brushing teeth for at least 30 min before all sample collections occurred at the riding center.

#### Randomization

Participants meeting inclusion criteria were then randomized into either an intervention group (THR) or control Barn Activity (BA) control group with no horse contact, stratified by NVIQ (≤85 or >85).

#### Screening Riding Center for THR Research Site

This replication trial took place at a therapeutic riding center, located in a rural setting in the foothills of northern Colorado, approximately 1-h driving time from Wyoming. This riding center has been operating since 1997 and maintained Premier Accreditation through PATH (Professional Association of Therapeutic Horsemanship) International since 2002. This premiere accreditation status is the highest level of accreditation in the field of equine assisted activities and therapies (EAAT) and requires the facility to follow rigorous and comprehensive standards across all aspects of programming, including safety and animal welfare. This facility has 23 acres, two indoor arenas, a large outdoor arena and a large sensory trail. The riding center was evaluated for appropriateness to conduct research based on a standardized site review. The research site review screening addressed the need for consistent, high quality programming for the duration of the 10-week intervention. During an on-site observation with research staff, the riding center confirmed it was able to provide an appropriate indoor/outdoor facility, horse's sound in mind and body, trained volunteers, and staff qualified to work with riders with ASD.

#### Screening Visit II: Riding Center

After participants' medical clearance forms were completed by and received from their physicians and caregivers, participants met with their assigned group leader at the riding center for an adaptive functioning screen. This screening visit involved an interview with the participant and caregiver about the participant's strengths and needs as well as a standardized 10-min direct observational assessment of the participant's adaptive skills. For the THR group this involved a 10-min horseback riding activity and for the BA control group, a drawing activity about horses.

#### Intervention Fidelity

Before initiating interventions, site riding center instructors and volunteers participated in a 2-h presentation reviewing methods for working with children with ASD in the riding center environment. This presentation was delivered by the on-site coordinator (second author, who was a certified Advanced PATH International therapeutic riding instructor). Prior to the intervention phase of this study, this coordinator also trained the two riding center THR group instructors on the manual-based (38) methods for conducting the 10-week THR intervention and provided on-site observation of instructor implementation of 20% of the THR lesson to measure intervention fidelity. BA control group instructor implementation of 20% of lessons were also observed and measured using this same fidelity tool by the senior author, who was 80% reliable with the on-site coordinator on three consecutive THR lessons (38).

#### Intervention and Control Groups

Both the 10-week THR and the BA control group intervention were 45-min in length and involved two to four participants, per group, with at least one volunteer assigned to assist per participant. The content of the THR and BA control groups were consistent for each of the 10-weekly lessons and included information about horses and horse care as described in the manual (38). However, the control group did not have interactions with horses, rather participants were only exposed to a pony-sized stuffed horse, which they used to practice activities such as grooming and tacking. Both groups were led by a THR instructor and employed teaching methods consistent with best practices for children with ASD that included use of consistent routines, visual schedules, demonstration and other concrete visual cues to enhance comprehension of information and expectations. Both the THR and control groups were (45 min in length and involved the following general schedule of routines:

- Saliva collection
- Sit with a volunteer
- Start group
- Review group schedule
- Warm up exercises
- Lesson & activity
- Cool down exercises
- THR group dismount & thank horses – All groups thank volunteers
- Drawing activity at table (20 min)
- Saliva collection

Of note, the control group leader and co-leader were the same as those who led the control group in the previous RCT (1). The THR and BA control groups occurred simultaneously (same day and afternoon times) at the riding center.

### Outcome Measures

#### Baseline and post-intervention Measures

##### Systematic Analysis of Language Transcripts (SALT)

Within one month pre- and post-THR and control group interventions, a study speech therapist blind to participants' condition group assignment conducted a five-minute language sample with each participant using the Systematic Analysis of Language Transcripts (SALT) (39). The SALT (39) provides standard guidelines to elicit, transcribe, and analyze language samples from individuals, including those diagnosed with ASD. Language samples were transcribed from recordings and then entered into the SALT language analysis program to compute vocabulary diversity. The SALT (39) was an outcome measure used and described in the previous RCT (1).

##### Social Responsiveness Scale (SRS)

Additionally, within 1 month pre- and post- interventions, a consistent caregiver for each participant completed the Social Responsiveness Scale (SRS) (18) about their child's social behaviors. The SRS measures social impairments of ASD that includes five subscales (Social Awareness, Social Cognition, Social Motivation, Social Communication and Autistic Mannerisms) (18). The SRS was an outcome measure also described in the previous RCT (1, 18).

#### Intervention Phase Measures

##### Aberrant Behavior Checklist–Community (ABC-C)

During the 10-week intervention phase of this study, the identified consistent caregiver for each participant completed the ABC-C (17) form to report on participant's behavior observed during the week preceding each group lesson (THR or control). The subscales of the ABC-C include Irritability, Lethargy/Social Withdrawal, Stereotypy, Hyperactivity, and Inappropriate Speech behaviors and items are rated on a 0-3 Likert-type severity rating scale. This is a 58-item symptom checklist was the primary outcome measure described and demonstrating significant changes in participants of the THR group from the previous RCT (1).

##### Saliva collection and determination of cortisol

Immediately before each THR session and 20 min following each session, study personnel collected saliva samples from participants (THR and control) using an absorbent swab specifically designed for use with children (SalivaBio, Carlsbad, CA). These collection times occurred at a consistent afternoon time (between 1:00-5:00 PM) when diurnal cortisol levels typically decline (40). The first sample was collected immediately before the groups when participants were seated with their volunteers either on a bench in the arena (THR group) or at a group table (BA control group). Participants were instructed to mouth the foam rod for 1 min. A mini 1-min sand timer was given to each participant to provide visual reference and enable them to track the collection time duration. The second saliva sample was collected 20 min after the conclusion of the standard 45-min THR or BA control group lessons (i.e., after dismounting the horse for the THR group and completing a review of things learned for the BA control group). Our methods to collect cortisol 20 minutes' post intervention is supported by previous findings that there is a 5-20-min lag in the detection of salivary cortisol (41). Participants followed the same procedures as previously described as each group participants sat at a table with their respective small groups and engaged in coloring or painting pictures. Each group (THR and control) sat in a separate room and did not have contact with each other. All samples were immediately frozen and shipped frozen to the Institute for Interdisciplinary Salivary Bioscience Research (IISBR) laboratory for analyses. Following methods described by Granger et al. (25), all saliva samples were assayed for cortisol using a commercially available immunoassay specifically designed for use with saliva without modification to the manufacturers recommended protocol https://www.salimetrics.com/assay-kits/#tab1 (Salimetrics, Carlsbad; Cat #1-3002). On the day of assay, samples were thawed, centrifuged to remove mucins, and assayed for cortisol in duplicate using an immunoassay specifically designed for use with saliva (Salimetrics, Carlsbad, CA) without modification to the manufacturers recommended protocol. The sample test volume was 25 μl, range of calibrators from 0.01 to 3.0 μg/dL, and lower limit of sensitivity 0.007 μg/dL. On average, inter and intra-assay coefficients of variation were less than 10 and 5% respectively. The average of the duplicate assays for each sample was used in the statistical analyses. Units for cortisol are expressed in micrograms per deciliter (ug/dL).

### Data Analysis

All the analyses were conducted using SAS 9.4 software (SAS Institute Inc.^1^). Demographic, diagnosis and baseline data were compared using Student *t*-tests and Fisher's exact tests for continuous and categorical variables respectively. The primary intent-to-treat analyses included data collected within 1 month pre- and post-THR and control group (or pre-session level of salivary cortisol at first and last week of intervention) and used a linear mixed effects model (LMM) without any data imputation. The LMM model consists of the baseline value and the post-evaluations as outcome measures, evaluation time (baseline or post-evaluation) of outcome, group (THR or control) and their interaction term as fixed effects and an unstructured covariance. Test of the time by group interaction term was used to assess the statistical significance of THR effectiveness. Effect size was calculated as (2xt value)/$\sqrt{\left(\text{DF}\right)}$, from the contrast of the time by group interaction. Sensitivity analyses were conducted to see how robust the conclusion were, including: (a) repeating the ITT primary analyses among participants completed at least 80% of THR or BA lessons, (b) testing the effectiveness using LMM model while adjusting for age and NVIQ and baseline anxiety score and (c) fitting a linear mixed model to all the weekly data of ABC-C (17) and testing the time by group interaction. Weekly immediate change in salivary cortisol level after an intervention lesson was compared between two groups using LMM model. Association of this immediate cortisol change with irritability and hyperactivity was examined using LMM model. The fidelity of the THR treatment implementation was computed as a percentage of the eight intervention component ratings. Irritability subscale of ABC-C (17) was deemed as the primary outcome. No adjustment for multiple secondary outcome variables was applied.

### Power of the Study

This study was a pilot study to replicate the RCT (1) study in a new riding center. This study was not powered to detect a specific effect size. A sample size of 16 (8 per arm) allows to detect an effect size of 1.5 common standard deviation with 80% power at 5% significance.

## Results

### Preliminary Analyses

Of the 17 potential participants screened, 16 (94%) met study inclusion criteria and were enrolled in this trial and randomized (see Figure 1). Of note, 75% of this sample had community-based psychiatric diagnoses. Every participant in THR group and four participants in control group had one or more psychiatric diagnoses. On the ABC-C (17) measure, participants in THR group had a more stereotypy behaviors and were more irritable and hyperactive at baseline. On the SCAS-P, participants in THR group had higher score on the Panic/Agoraphobia subscale. The groups did not differ otherwise at baseline (see Tables 1, 2). Five THR participants completed all 10 THR lessons; two completed nine lessons; and one completed five lessons. Two BA control group participants completed all 10 intervention lessons, four completed nine lessons, one completed eight lessons and one completed one lesson.

**Table 1:** Characteristics of Participants.

**Characteristic**	**THR**	**BA control**	***p*-value**^**a**^
Number of participants	8	8	
Age, (Mean (SD), years)	11.88 (2.45)	9.80 (2.82)	0.14
Gender, males/females (counts)	6/2	7/1	1.0
IQ (Mean (SD)	102.88(16.28)	100.25 (29.26)	0.83
**SCAS-P**			
Panic Agoraphobia	4.63 (3.50)	1.13 (1.25)	0.03
Separation Anxiety	7.63 (5.76)	4.38 (4.14)	0.22
Physical Injury Fears	5.50 (4.04)	3.13 (3.04)	0.21
Social Phobia	5.50 (3.59)	3.38 (3.34)	0.24
Obsessive Compulsive	4.38 (4.07)	1.63 (1.51)	0.11
Generalized Anxiety Overanxious	6.63 (4.84)	3.88 (3.83)	0.23
Community psychiatric diagnoses Y/N (counts)	8/0	4/4	0.08
Current seizure disorder, Y/N (counts)	0/8	0/8	1.0
Psychotropic medicine, Y/N (counts)	6/2	3/5	0.31
Psychotic disorder	1/7	0/8	1.0
Mood disorder, Y/N (counts)	3/5	0/8	0.2
Anxiety disorder, Y/N (counts)	5/3	3/5	0.62
ADHD, Y/N (counts)	5/3	2/6	0.31
Learning disability, Y/N (counts)	1/7	0/8	1.0
Latino/Hispanic	1/7	0/8	1.0
Race			1.0
Caucasian	8	7	
Multiracial		1	

a *Two tailed p-value from two sample t-test and Fisher's exact test as appropriate (ug/dL). Date points of same symbol are from the same participants*.

**Table 2A:** Analysis of efficacy of Therapeutic Horseback Riding (THR) (*n* = 8) compared to the Barn Activity (BA) control (*n* = 8)^a^.

	**THR Group**			**BA Control Group**			**Interaction (Efficacy)**		
	**Baseline Mean (SD)**	**EoT Mean (SD)**	**Change Mean (SEM)**	**Baseline Mean (SD)**	**EoT Mean (SD)**	**Change Mean (SEM)**	**Mean (SEM)**	***p***^**d**^	**ES**^**c**^
**PRIMARY OUTCOME VARIABLE – ABC-C**									
Irritability^b^	21.75 (13.27)	14.43 (13.38)	−6.44 (4.71)	11.57 (5.56)	18.33 (10.86)	6.97 (5.33)	−13.42 (7.11)	0.08	1.08
Lethargy	14.63 (9.59)	10.71 (3.20)	−4.64 (3.02)	10.57 (4.76)	11.83 (9.85)	2.29 (3.44)	−6.93 (4.58)	0.16	0.91
Stereotypy	6.38 (4.00)	5.29 (4.39)	−0.59 (0.77)	5.29 (6.16)	5.33 (6.80)	0.70 (0.92)	−1.29 (1.20)	0.31	0.67
Hyperactivity	22.50 (12.15)	16.00 (8.64)	−5.90 (3.28)	17.14 (4.10)	24.33 (6.02)	7.40 (3.73)	−13.30 (4.97)	**0.02**	1.49
Inappropriate Speech	4.50 (1.51)	3.43 (2.23)	−0.95 (1.16)	4.86 (3.58)	4.67 (4.03)	0.34 (1.32)	−1.29 (1.75)	0.48	0.43
**SECONDARY OUTCOME VARIABLE - SRS**									
Social awareness	14.63 (4.31)	11.29 (1.38)	−3.67 (1.27)	12.38 (2.39)	13.57 (4.12)	1.23 (1.27)	−4.90 (1.80)	**0.02**	1.54
Social cognition	19.50 (7.09)	21.29 (3.30)	1.25 (1.85)	16.75 (6.36)	18.71 (7.43)	1.90 (1.85)	−0.66 (2.61)	0.81	0.14
Social Communication	37.38 (13.41)	34.57 (3.95)	−5.20 (2.48)	30.75 (10.00)	31.29 (10.98)	1.50 (2.48)	−6.70 (3.51)	0.08	1.17
Autistic Mannerism	20.38 (6.76)	20.29 (4.96)	−0.77 (2.17)	17.13 (4.76)	18.86 (6.47)	1.65 (2.17)	−2.42 (3.07)	0.45	0.45
Social Motivation	16.88 (5.99)	16.43 (4.28)	−1.96 (1.07)	13.25 (5.73)	12.71 (6.05)	−0.06 (1.07)	−1.90 (1.51)	0.23	0.73
**SECONDARY OUTCOME VARIABLE - SALT**									
Number different words	143.75 (65.40)	152.50 (66.06)	8.18 (11.01)	108.88 (81.04)	107.14 (57.03)	−12.96 (10.40)	21.14 (15.15)	0.19	0.78
Number words used	343.00 (171.53)	372.17 (170.43)	28.22 (30.85)	252.50 (208.57)	237.86 (138.22)	−39.86 (29.32)	68.07 (42.56)	0.13	0.88
**SECONDARY OUTCOME VARIABLE - SALIVARY CORTISOL LEVEL (ug/dL)**									
Pre-session cortisol	0.12 (0.084)	0.13 (0.09)	0.007 (0.04)	0.13 (0.08)	0.10 (0.07)	−0.03 (0.04)	0.037 (0.05)	0.49	0.41
Post-session cortisol	0.05 (0.02)	0.06 (0.03)	0.008 (0.04)	0.09 (0.05)	0.13 (0.13)	0.034 (0.04)	−0.026 (0.05)	0.63	0.29

a *Analyses included all participants who were randomized and had either baseline line and/or End of treatment (EoT) assessment. Cortisol assessed at intervention weeks one and the last week (weeks 9 or 10) for THR (n = 7) and BA control (n = 7) groups were used to approximate baseline and EoT cortisol level. Sample means and standard deviation were reported for baseline and EoT. Mean and standard errors of change and the time by group interaction are from mixed effects model analysis of baseline and EoT data for all the outcome variables. The mixed effects model consists of time (baseline/EoT), group (THR/BA control) and their interaction as fixed effects and an unstructured covariance. Test of the time by group interaction (i.e., THR minus Barn control in change from baseline) is used to assess the efficacy of THR*.

b *Irritability subscale is deemed as the primary efficacy outcome in this study*.

c *Effect size is calculated $\left(2\times t\;value\right)/\sqrt{DF}$ from the contrast of the time by group interaction*.

d *p-value < 0.05 are in bold face form*.

### Intervention Fidelity

#### THR Group

The average overall fidelity rating for the THR group was 92.22%, with average ratings in the four domains as follows: Teaching Techniques & Class Structure 88.32%; Volunteers 100%; Environment 100%.

#### Control Group

The average overall fidelity rating for the control group was 93.47%, with average ratings in the four domains as follows: Teaching Techniques & Class Structure 95.37%; Volunteers 80.55%; Environment 95.83%.

## Clinical Outcomes

**Figure 2:**
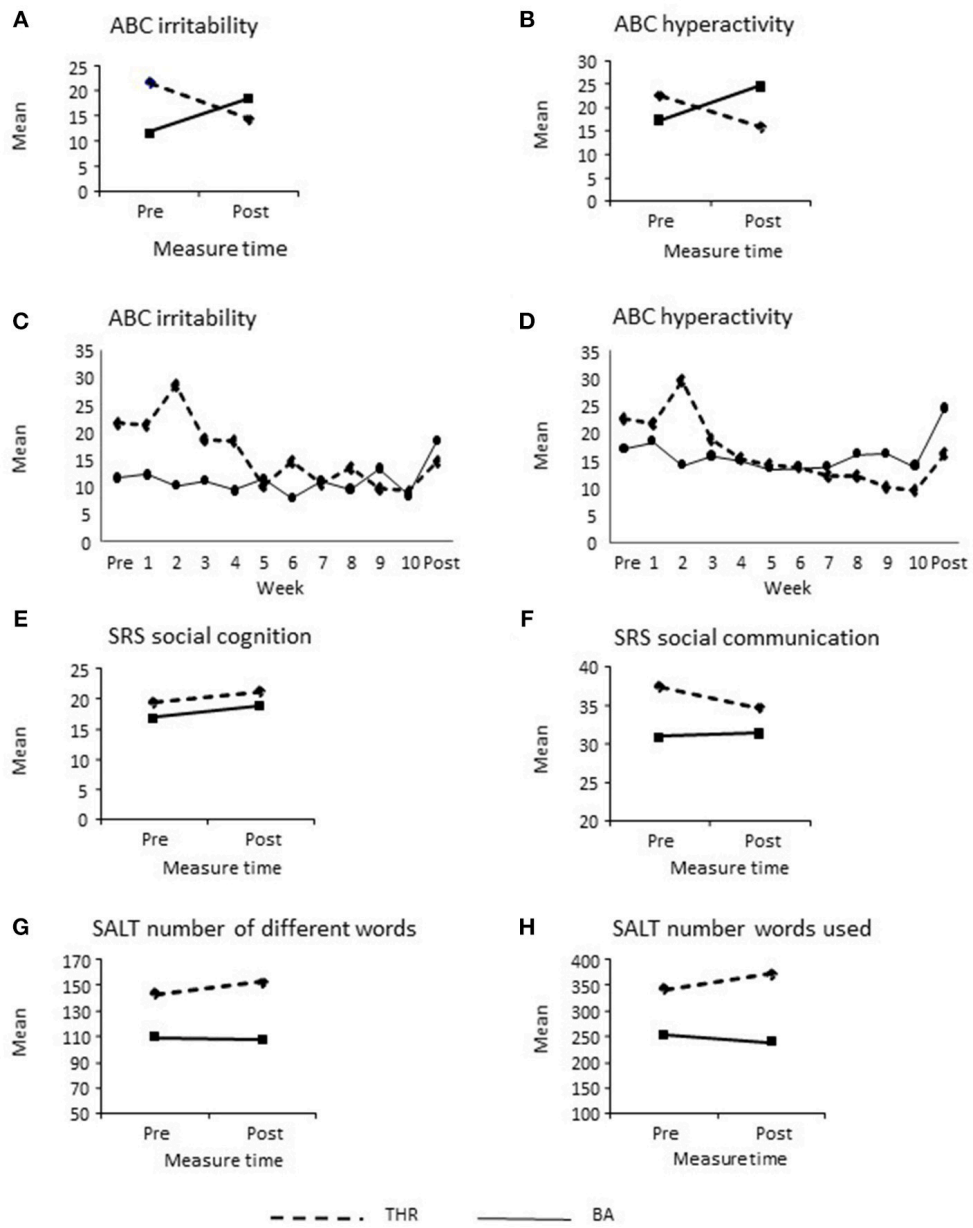
**(A–H)** Efficacy of the THR intervention compared to control on primary and secondary outcome variables.

Tables 2A,B show the effectiveness of the THR intervention compared to the BA control group for the primary (ABC-C) and secondary (SRS, SALT, salivary cortisol) outcome variables. Figure 2 shows the mean response patterns of the six outcome variables on which THR demonstrated favorable effect from the original RCT (1).

**Table 2B:** Completer analysis for efficacy^a^.

	**THR (*****n*** **=** **7)**			**BA Control (*****n*** **=** **7)**			**Interaction**		
	**Baseline Mean (SO)**	**EoT Mean (SO)**	**Change Mean (SEM)**	**Baseline Mean (SO)**	**EoT Mean(SO)**	**Change Mean (SEM)**	**Mean (SEM)**	***p***^**d**^	**ES**^**c**^
**ABC-C**									
lrritability^b^	19.86 (13.12)	14.43 (13.38)	−5.43 (4.74)	12.00 (5.97)	18.33 (10.86)	6.76 (5.39)	−12.19 (7.18)	0.12	−1.00
Lethargy	16.57 (8.48)	10.71 (3.20)	−5.86 (2.96)	10.00 (4.94)	11.83 (9.85)	2.65 (3.38)	−8.50 (4.49)	0.08	−1.14
Stereotypy	5.86 (4.02)	5.29 (4.39)	−0.57 (0.76)	4.83 (6.62)	5.33 (6.80)	0.74 (0.91)	−1.31 (1.19)	0.30	−0.69
Hyperactivity	20.86 (12.13)	16.00 (8.64)	−4.86 (3.37)	17.33 (4.46)	24.33 (6.02)	7.28 (3.86)	−12.14 (5.12)	**0.04**	−1.39
Inappropriate Speech	4.29 (1.50)	3.43 (2.23)	−0.86 (1.15)	4.33 (3.61)	4.67 (4.03)	0.62 (1.32)	−1.48 (1.75)	0.42	−0.50
**SRS**									
Social awareness	15.43 (3.95)	11.29 (1.38)	−4.14 (1.27)	12.29 (2.56)	13.57 (4.12)	1.29 (1.27)	−5.43 (1.80)	**0.01**	−1.74
Social cognition	20.43 (7.11)	21.29 (3.30)	0.86 (1.90)	16.86 (6.87)	18.71 (7.43)	1.86 (1.90)	−1.00 (2.68)	0.72	−0.22
Social Communication	41.00 (9.33)	34.57 (3.95)	−6.43 (2.35)	29.29 (9.83)	31.29 (10.98)	2.00 (2.35)	−8.43 (3.32)	**0.03**	−1.46
Autistic Mannerism	21.71 (6.05)	20.29 (4.96)	−1.43 (2.16)	17.29 (5.12)	18.86 (6.47)	1.57 (2.16)	−3.00 (3.06)	0.35	−0.57
Social Motivation	18.57 (3.87)	16.43 (4.28)	−2.14 (1.05)	12.71 (5.96)	12.71 (6.05)	−0.00 (1.05)	−2.14 (1.49)	0.18	−0.83
**SALT**									
Number different words used	142.14 (70.47)	152.50 (66.06)	8.57 (11.15)	123.71 (74.88)	107.14 (57.03)	−16.57 (10.55)	25.14 (15.35)	0.13	0.95
Number words used	340.00 (185.05)	372.17 (170.43)	29.08 (31.59)	287.86 (197.69)	237.86 (138.22)	−50.00 (30.10)	79.08(43.63)	0.09	1.04

a *Analyses included all participants who were randomized and had either baseline line and/or End of treatment (EoT) assessment among those completed 80% of intervention lessons. Sample means and standard deviation were reported for baseline and EoT. Mean and standard errors of change and the time by group interaction are from mixed effects model analysis of baseline and EoT data for all the outcome variables. The mixed effects model consists of time (baseline/EoT), group (THR/Barn activity control) and their interaction as fixed effects and an unstructured covariance. Test of the time by group interaction (i.e. THR minus Barn in change from baseline) is used to assess the efficacy of THR*.

b *Irritability subscale is deemed as the primary efficacy outcome in this study*.

c *Effect size is calculated $\left(2\times t\;value\right)/\sqrt{DF}$ from the contrast of the time by group interaction*.

d *p-value < 0.05 are in bold face form*.

### Primary Outcome Variable (ABC-C)

Participants in the THR group had lower average post- treatment Irritability and Hyperactivity subscale scores while participants in BA control group had higher average post-treatment scores for both subscales as compared to the baseline values (see Figures 2A,B). Between-treatment difference in post-treatment change was significant on the Hyperactivity subscale (es = 1.49, *p* = 0.02) and significant at the 0.1 significance level on the Irritability subscale (es = 1.08, *p* = 0.08), indicating THR participants made more improvements from baseline to post-treatment on both outcomes compared to BA control group participants. Moreover, a consistent result was found from the LMM analysis with baseline panic agoraphobia score as a covariate for irritability (*p* = 0.09) and hyperactivity (*p* = 0.02). Although not statistically significant, larger panic agoraphobia score was associated with larger irritability, but small hyperactivity score. If age and non-verbal IQ were adjusted in the LMM model, significant effectiveness of THR was found respectively for irritability (*p* = 0.037) and hyperactivity (*p* = 0.013). The time course of the weekly Irritability and Hyperactivity scales (see Figures 2C,D) were also analyzed using a linear mixed effects model (LMM). Statistical test of the time by treatment interactions were significant (*p* = 0.016 for the Irritability and *p* = 0.0005 for Hyperactivity subscales). For the Irritability subscale, baseline and post-treatment means (SEM) estimated by LMM were respectively 21.75 (3.88) and 16.19 (3.96) for THR participants and 10.47 (3.95) and 17.28 (4.05) in BA control group participants, resulting in the between-treatment difference in change from baseline of 12.36 (3.97), which was statistically significant (*p* = 0.0023). For the Hyperactivity subscale, baseline and post-treatment means (SEM) were respectively 22.5 (3.03) and 17.67 (3.11) for THR participants and 16.22 (3.10) and 24.82 (3.19) in the BA control group participants; the corresponding between-treatment difference in change from base was then 13.42 (3.47), which was statistically significant (*p* = 0.0002). There was no significant difference between the two groups on any of the other ABC-C (17) subscales.

To examine the robustness of these primary analyses, the same analysis was repeated among the THR (*n* = 7) and BA (*n* = 7) participants, each who completed 80% or more intended sessions. These produced the same results for effects on the Irritability and Hyperactivity subscales (Table 2A).

### Secondary Outcome Variables SRS, SALT, and Salivary Cortisol

#### SRS

For the SRS (18), the THR group had greater improvements on the Social Communication (*p* = 0.08) and Social Awareness (*p* = 0.02) subscales compared to the BA control group. In analysis of participants who completed at least 8 weeks of the THR and BA control group interventions, the SRS Social Communication subscale became significant (*p* = 0.03), a finding similar to the previously published RCT (1). There was no significant difference between groups on any other of the SRS subscales.

#### SALT

On the SALT (39) there was no statistically significant difference in improvement of number words or different words spoken after treatment between the two groups even though the response pattern was in favor of THR group, similar to the previous RCT (1).

#### Salivary Cortisol

We compared week one cortisol levels and the cortisol levels collected the last week of intervention to assess efficacy. Separate analyses were conducted for pre-lesson and post-lesson cortisol levels. Median (range) of salivary sample collection times of pre-lesson were 13:45 (12:53–13:45) for THR and 13:45 (12:15–14:30) for BA at first week and 14:23 (12:50–14:30) for THR and 12:30 (12:27–14:09) for BA at the last lesson. There was no difference between two groups in the change of pre-lesson (*p* = 0.49) or post-lesson (*p* = 0.63) cortisol levels between the first and last week of the intervention (Table 2A). This non-significance remained after adjusting for salivary sampling time and baseline panic agoraphobia scores (*p* = 0.61 for pre-lesson and *p* = 0.62 for post-lesson cortisol). Of a total of 60 completed THR lessons, pre- and post-lesson salivary samples were successfully collected for 90% riding lessons while salivary samples were collected in 74% of a total 65 BA control lessons.

Looking at all the weekly data together with a LMM analysis, a significant decrease in cortisol after the THR lessons was observed in THR participants (mean (SEM): from 0.11 (0.012) to 0.07 (0.009), *p* = 0.004). The decrease in cortisol after the BA control lessons was significant at 0.1 level in the BA control participants (mean (SEM): from 0.13 (0.014) to 0.10 (0.010), *p* = 0.07). However, THR group did not show significantly more post-lesson decline in cortisol as compared to BA control (*p* = 0.38).

**Figure 3:**
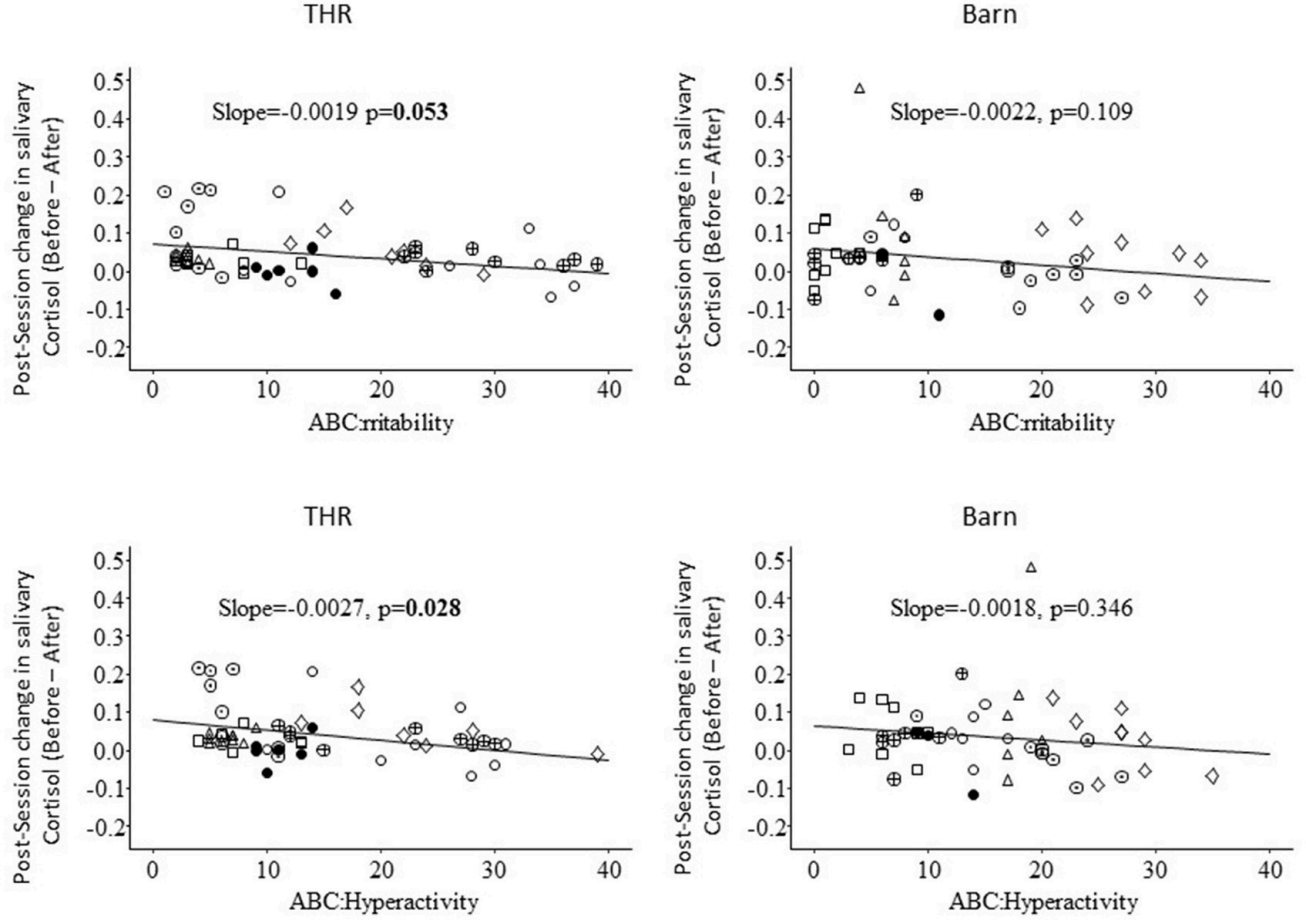
Association of ABC-C Irritability and Hyperactivity with immediate post-lesson THR or BA control group change in salivary cortisol.

In fact, the post-lesson cortisol change can either be an increase or decrease, varying from participant to participant from week to week. Association of weekly Irritability or Hyperactivity subscale scores with weekly post-lesson cortisol change was then examined using LMM. Greater ABC-C weekly Irritability and Hyperactivity scores were respectively associated with a smaller amount of cortisol reduction after the THR lesson (Figure 3, slope = 0.002, *p* = 0.053 for Irritability and slope = 0.003, *p* = 0.028 for Hyperactivity). Such a relationship was not statistically significant in BA the control group. However, there was no statistically significant difference in slope between the two groups (*p* = 0.68 for Irritability and *p* = 0.93 for Hyperactivity). These LMM analyses were also conducted while adjusting for time of pre-lesson salivary cortisol sampling and the minutes between pre- and post-lesson salivary cortisol collection in order to remove the potential confounding effect of the diurnal decrease of cortisol. These analyses produce the same significant (*p* < 0.05) correlation results as the unadjusted analyses.

## Discussion

This article reports results from a replication pilot of an RCT study that evaluated the effects of THR for children with ASD (1). Both studies compared a 10-week manual-based (38) THR intervention to a BA control group. The present replication study took place at a different riding center and enrolled 16 participants ages 6–16 years with a study confirmed diagnosis of ASD. The goals of the current study were to replicate the RCT, and to explore the effect of THR on salivary cortisol for children with ASD. Part of the results of the RCT were replicated, in that compared to the BA control group, THR participants significantly improved on the ABC-C (17) Hyperactivity subscale (*p* = 0.02). Additionally, the THR group had significant improvements at 0.1 level on the ABC-C (17) Irritability subscale (*p* = 0.08) and SRS (18) Social Communication subscale (*p* = 0.08). The replication of finding for hyperactivity but not the irritability subscale on the ABC-C matches up with another small scale study of the effect of THR for children with ASD (19), indicating that THR may have a stronger effect on hyperactivity than on irritability behaviors. There were no significant improvements in the number of words or new words spoken on the SALT (39) standard language sample. There was no significant decrease in salivary cortisol over 10-weeks intervention for either the THR or the BA control group. When examining the immediate pre- and post-lesson cortisol level changes, children with lower pre-session measures of Hyperactivity and Irritability behaviors on the ABC-C showed greater post-lesson decreases in salivary cortisol. This may suggest that pre-lesson cortisol can be considered as target mediator outcome for future THR research.

There are several limitations of this study. This study is limited by the small sample size, which limited power and randomization. Although randomly assigned, groups were significantly different from one another in pre-test irritability and hyperactivity, and co-occurring conditions. This factor may lead to a biased estimate of THR efficacy due to a regression to the mean. The THR intervention was replicated in the same state where the original trial was conducted, which limits the generalization of the results to other populations.

This is the first known study to report partial replication of results from a previous RCT of THR, thereby extending previous THR efficacy findings by examining the effects of a standardized THR intervention at a different riding center. A future larger scale replication study can provide conclusive replication validation. This study also provides preliminary data to objectively evaluate if the act of riding a horse in the context of a standard 10-week THR group can have immediate biological effect on reducing stress levels as measured by salivary cortisol levels as compared to the BA control. Although significant between group differences on cortisol reduction was not found in this pilot study, it appears that the extent of cortisol reduction after THR was associated with the participants' level of irritability and hyperactivity prior to riding. This very preliminary finding suggests that cortisol may play some role in the THR effect on irritability and hyperactivity. A larger scale study is required to investigate the potential mediation effect of cortisol activity on THR.

## Ethics Statement

This study was carried out in accordance with the recommendations of the Colorado Multiple Institutional Review Board. The protocol was approved by the Colorado Multiple Institutional Review Board. All subjects gave written informed consent in accordance with the Declaration of Helsinki. This study protocol was approved by the Colorado Multiple Institute Review Board.

## Author Contributions

ZP served as the statistical expert for this study and wrote the result section of this manuscript. DG assisted with study design and methods, supervised assay of project samples, and provided editorial comments on the manuscript. NG assisted in writing the introduction of this manuscript. AS was contracted as consultant for this study and provided editorial edits to this manuscript. RG was the principal investigator of this study and contributed to writing the majority of this manuscript.

### Conflict of Interest Statement

RG is a co-author of the book, Growing Up with Autism: Working with School-aged Children and Adolescents (Guilford Press) and the book, Autism from Research to Individualized Practice (Jessica Kingsley Publishers), from which she receives royalties. Current grant funding for RG provided by Simons and Lurie Foundations, MARS/WALTHAM, and The Human-Animal Bond Research Institute (HABRI) Foundation. We note that DG is the founder and Chief Scientific and Strategy advisor at Salimetrics and Salivabio and the nature of these relationships is managed by the policies of the committees on conflict of interest at Johns Hopkins University School of Medicine and the University of California at Irvine The remaining authors declare that the research was conducted in the absence of any commercial or financial relationships that could be construed as a potential conflict of interest.

## Footnotes

^1^SAS Institute Inc., “SAS.” (Cary, NC).
